# Renal microvasculature in the adult pipid frog, *Xenopus laevis*: A scanning electron microscope study of vascular corrosion casts

**DOI:** 10.1002/jmor.21132

**Published:** 2020-05-06

**Authors:** Alois Lametschwandtner, Bernd Minnich

**Affiliations:** ^1^ Department of Biosciences University of Salzburg, Vascular and Exercise Biology Research Group Salzburg Austria

**Keywords:** kidney, scanning electron microscopy, vascular casts, *Xenopus*

## Abstract

We studied the opisthonephric (mesonephric) kidneys of adult male and female *Xenopus laevis* using scanning electron microscopy (SEM) of vascular corrosion casts and light microscopy of paraplast embedded tissue sections. Both techniques displayed glomeruli from ventral to mid‐dorsal regions of the kidneys with single glomeruli located dorsally close beneath the renal capsule. Glomeruli in general were fed by a single afferent arteriole and drained via a single thinner efferent arteriole into peritubular vessels. Light microscopy and SEM of vascular corrosion casts revealed sphincters at the origins of afferent arterioles, which arose closely, spaced from their parent renal arteries. The second source of renal blood supply via renal portal veins varied interindividually in branching patterns with vessels showing up to five branching orders before they became peritubular vessels. Main trunks and their first‐ and second‐order branches revealed clear longish endothelial cell nuclei imprint patterns oriented parallel to the vessels longitudinal axis, a pattern characteristic for arteries. Peritubular vessels had irregular contours and were never seen as clear cylindrical structures. They ran rather parallel, anastomosed with neighbors and changed into renal venules and veins, which finally emptied into the ventrally located posterior caval vein. A third source of blood supply of the peritubular vessels by straight terminal portions of renal arteries (vasa recta) was not found.

## Graphical abstract

Scanning electron microscopy of vascular corrosion casts of kidneys of adult *Xenopus laevis* convincingly confirms that renal portal veins supply most of the renal parenchyma and renal arteries primarily feed renal glomeruli.

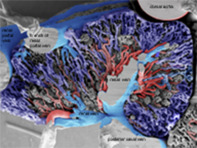

## 1 INTRODUCTION

Kidneys conserve body fluids and electrolytes (osmoregulation), remove metabolic waste and function also as endocrine organs (Ross, Kaye, & Pawlina, 2003). Nephrons and the blood vascular system are the main functional components. Both systems are particularly well studied in mammals and men. Scanning electron microscopy (SEM) of vascular corrosion casts is a powerful technique to visualize the renal microvascular anatomy. It brought detailed insights into the three‐dimensional vascular architecture of the entire kidneys as well as of single glomeruli (e.g., rat: Murakami, 1971, 1972, 1976; men: Murakami, Kikuta, Akita, & Sano, 1985; Kikuta & Murakami, 1989). These studies convincingly demonstrated that renal glomeruli consist of interconnected wide capillaries, which form several lobules with, in general, one afferent and one efferent arteriole. Occasionally, multiple efferent arterioles were found in men (Murakami et al., 1985), and double afferent and efferent arterioles in rats were described by Murakami (1971, 1976) as well.

A comparative study by Ditrich and Splechtna (1990) gave an impressive overview on glomerular size and shape from members of all vertebrate classes. The authors pointed out that cyclostomes (*Myxine glutinosa*: Albrecht, Lametschwandtner, & Adam, 1978) have the largest glomeruli (the average of equatorial diameter: ~875 μm), while birds (*Gallus domesticus*) have the smallest ones (equatorial diameter: ~60 μm).

To date, the histomorphology of kidneys of adult ranids and bufonids has been well documented (*Rana esculenta*, *Rana fusca*: Gaupp, 1899; Richter, 1995; *Rana cancrivora*; Uchiyama, Murakami, Yoshizawa, & Wakasugi, 1990; *Rana catesbeiana*: Rheubert, Cook, Sigel, & Trauth, 2017; *Bufo bufo*: Möbjerg, Larsen, & Jespersen, 1998; *Bufo arenarum*: Farias, Fiorita, & Hermida, 1998; Farias, Hermida, & Fiorita, 2003). That of adult *Xenopus laevis* was dealt with as a chapter in the color atlas on the overall *Xenopus* histology (Wiechmann & Wirsig, 2003).

Studies on the renal microvascular anatomy in adult anurans using SEM of vascular corrosion casts are still few and are limited to *B. bufo*, *Bombina variegata*, *Rana ridibunda*, and *X. laevis* (Lametschwandtner, Albrecht, & Adam, 1978), *Bufo marinus* (Morris & Campbell, 1978), and *R. catesbeiana* (Ohtani & Naito, 1980). Only two studies focused on the glomerular development and growth of the renal blood vascular system in *R. catesbeiana* (Naito, 1984) and *X. laevis* (Ditrich & Lametschwandtner, 1992).

In our previous study (Lametschwandtner et al., 1978), we primarily compared the overall renal microvascular anatomy of a ranid species (*R. ridibunda*), a bufonid species (*B. bufo*), a bombinid species (*B. variegata*), and a pipid species (*X. laevis*), but did not go into greater detail. As *X. laevis* becomes increasingly used also as a model to study basic renal development and repair (Chan & Asashima, 2006; Droz & McLaughlin, 2017; Lienkamp, 2016), a more detailed knowledge of its renal microvasculature may be helpful for future renal studies.

Our study gives an in‐depth analysis of the microvascular anatomy of the adult *Xenopus* kidney using SEM of vascular corrosion casts. It further tests if the distal portions of the renal arteries form straight arterioles (arteriolae rectae). These arterioles were described earlier to directly join peritubular capillaries and to establish a third source of blood supply to the peritubular capillaries. SEM of renal vascular casts can possibly answer whether the aforementioned arteriolae rectae described by Gaupp (1899) in *R. esculenta* also exist in *X. laevis*.

## 2 MATERIALS AND METHODS

### 2.1 Animals

Twelve adult animals of both sexes of the pipid frog, *X. laevis* were studied. Adults were housed in aquaria (tap water depth: 15 cm) equipped with aquarium filters and fed twice a week with either dried *Gammarus pulex* or grinded beef heart. The study was approved by the Ethics Committee of the University of Salzburg, Austria and the Federal Government (BMBWK‐66.012/0018‐BrGT/2006).

### 2.2 Histomorphology

Two animals (female: body weight: 79 g, body length: 9 cm; male: 79 g, body length: 9 cm) were euthanized by immersion into an aqueous solution of MS 222 (0.5%). After weighing, the heart was exposed, the sinus venous opened and animals were rinsed (Amphibian Ringer solution; Adam & Czihak, 1964) and fixed (Bouin's solution) via the truncus arteriosus. The flow rate of the infusion pump (Habel, Vienna) was set to 40 ml/hr. Fixed kidneys were excised, dehydrated, and embedded in paraplast. The kidneys of one individual were cut transversely, those of the second animal longitudinally and horizontally (thickness of sections: 7 μm). Sections stained according to Goldner (Adam & Czihak, 1964) were analyzed with an Olympus BX51 light microscope. Images were recorded by a digital camera (Olympus SC 50; Japan) attached to the microscope using Cell Sense Imaging software (Olympus). If necessary brightness and contrast of images were adjusted using Photoshop 7.0 (Adobe Inc., Redwood, CA).

### 2.3 Vascular corrosion casting

Ten adult animals (six males: body weights: 34–70 g, body lengths: 75–85 mm: four females: body weights: 39–203 g, body lengths: 60–115 mm) were studied. For euthanasia and rinsing, see “Section 2.2” described above. When clear reflux drained from the opened sinus venosus 10 ml of Mercox CL‐2B (Dainippon Ink and Chemicals, Tokyo, Japan; Ladd Burlington, Vermont, USA) diluted with monomeric methyl methacrylate (4 + 1, vol + vol, 10 ml monomeric methylacrylate contained 0.85 g initiator paste MA) were injected with the infusor at a flow rate of 41 ml/hr. When the effluent resin became viscous or the whole amount of resin had been perfused the injection was stopped and the animals were left for about 30 min at room temperature to allow hardening of the injected resin. After tempering the injected resin by placing the animals into a water bath (60°C; 12–24 hr), specimens were macerated in potassium hydroxide (7.5%; 40°C; 2–24 hr). Thereafter, rinsed three times in distilled water, submerged in 2% hydrochloric acid, rinsed three times in distilled water followed by submersion in formic acid (5%; 20°C; 5–15 min) to remove any residual organic matter from the cast surfaces. Finally, specimens were rinsed another three times in distilled water and frozen in fresh distilled water. Ice‐embedded casts were freeze‐dried in a Lyovac GT2 (Leybold‐Heraeus, Cologne, Ger). Casts of kidneys with testes in situ were excised and mounted onto specimen stubs using the “conductive bridge‐method” (Lametschwandtner, Miodonski, & Simonsberger, 1980). Specifically, thin copper wires were fixed from the cast to the specimen stub by colloidal silver to reduce specimen charging. These “conductive bridges” are marked by asterisks if displayed on SEM micrographs. Mounted specimens were either evaporated with carbon and gold and/or sputter‐coated with gold, and examined in the SEM XL‐30 (FEI, Eindhoven, The Netherlands) at an accelerating voltage of 10 kV.

In some specimens, course, branching patterns and areas of supply (or drainage) of individual renal vessels were exposed by ripping‐off overlaying vessels with fine tipped insect pins under binocular control. Sectioning of ice‐embedded vascular casts was done as described elsewhere (Lametschwandtner & Lametschwandtner, 1992).

Arteries and veins were distinguished by their characteristic endothelial cell nuclei imprint patterns displayed on the surfaces of vascular casts (Miodonski, Hodde, & Bakker, 1976). Color‐coding of arteries (red) and veins (blue) was done in Photoshop 7.0 (Adobe Inc.).

### 2.4 Two‐dimensional and three‐dimensional morphometry

Linear (two‐dimensional) measurements were taken from paraffin‐embedded tissue sections using an Olympus BX 51 microscope equipped with a digital camera and CellSense imaging software (Olympus).

**FIGURE 1:**
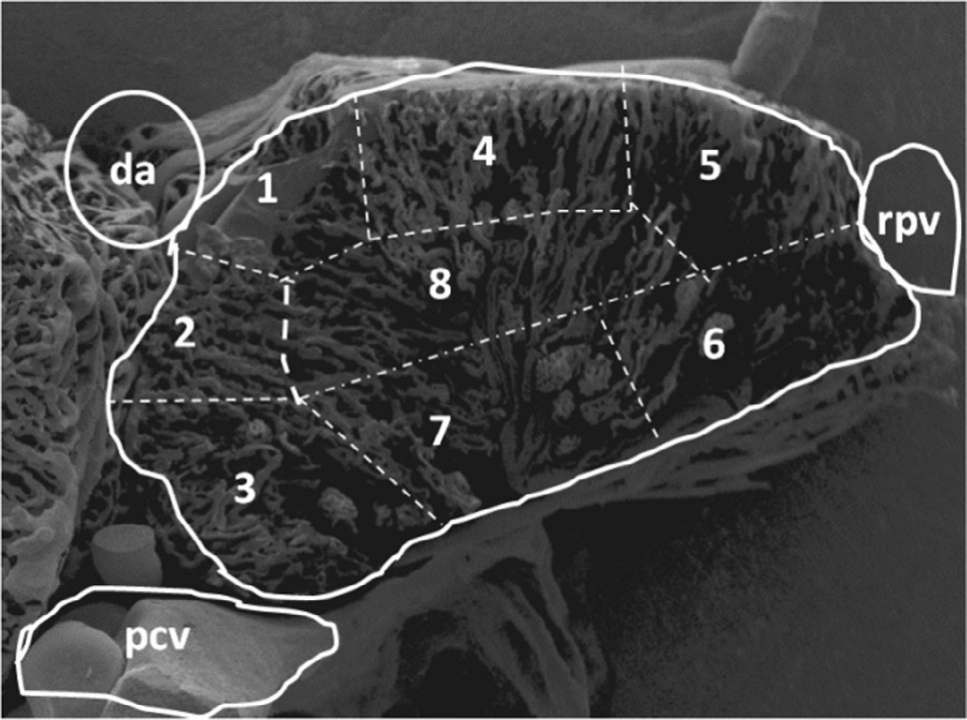
*Xenopu*s *laevis*, right kidney. Vascular cast. Transverse section displaying areas 1–8 where 3D‐morphometric measurements were taken. da, dorsal aorta; pcv, posterior caval vein; rpv, renal portal vein

For three‐dimensional (3D) morphometry of vascular corrosion casts stereo paired images (tilt angle 6°, working distance 10 mm) were recorded in the SEM (ESEM XL‐30; FEI) and imported into the M3‐software (ComServ OG, Ebenau, Austria; Minnich, Leeb, Bernroider, & Lametschwandtner, 1999, Minnich & Lametschwandtner, 2000). In detail, equatorial diameters and urinary‐vascular pole distances of glomeruli as well as diameters of afferent and efferent glomerular arterioles were measured within eight areas (see Figure 1) of eight transverse sections of renal casts from two male animals (body lengths: 80 and 85 mm). Data were subjected to descriptive statistical analysis (SigmaPlot 13; Systat Software GmbH).

## 3 RESULTS

**FIGURE 2:**
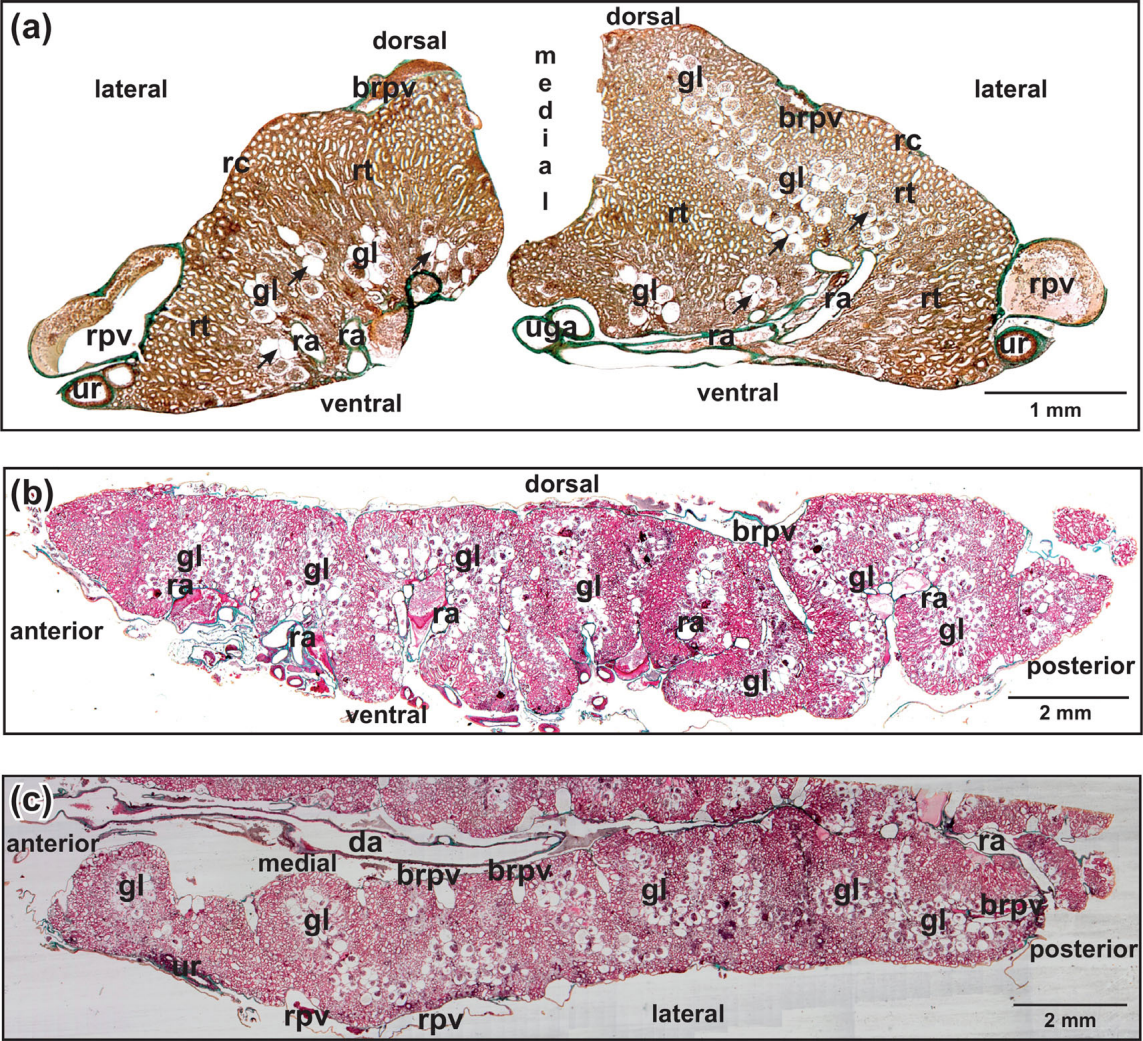
*Xenopus laevis*, histomorphology of kidneys of adult individual. Overview. Goldner staining. (a) Transverse section (thickness: 7 μm). Note the difference in size between right and left kidneys. Glomeruli of rather equal size are located from ventral to mid‐dorsal regions and are grouped in nests and rows (arrows). (b) Longitudinal section. Note that in posterior regions glomeruli are located close to the dorsal surface. (c) Horizontal section. Note that glomeruli are aligned from medial to lateral edges. Abbreviations: brpv, branch of renal portal vein; da, dorsal aorta; gl, glomerulus; ra, renal artery; rc, renal capsule; rpv, renal portal vein; rt, renal tubule; uga, urogenital artery; ur, ureter

The kidneys of adult *X. laevis* owned a capsule of thin connective tissue, contained numerous nephrons, a dense network of blood vessels, little stromal connective tissue between blood vessels, renal tubules, nerve fibers, and peritoneal funnels and tubules. Locally, accumulations of lymphocytes were present. Nephrons consisted of glomerulus, Bowman's capsule, and renal tubule. Dorsoventrally, glomeruli were located primarily within the ventral to mid‐dorsal regions (Figure 2a,b). Horizontally, they were found from lateral to medial (Figure 2c). Diameters of Bowman's capsules ranged from 150 to 210 μm, that is, they were much larger than those of glomeruli (average diameter: 150 μm).

**FIGURE 3:**
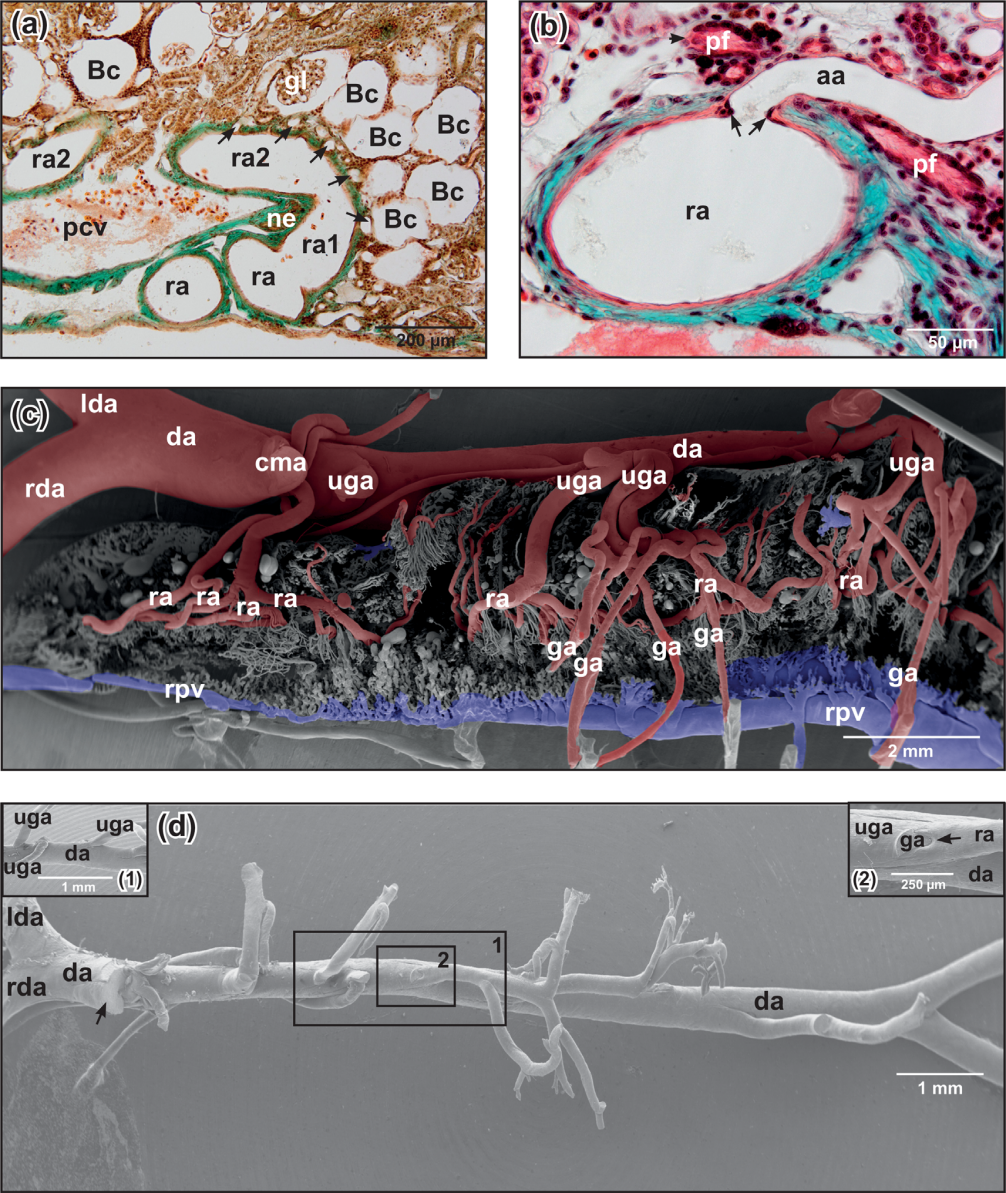
*Xenopus laevis*, histomorphology and microanatomy of renal blood vessels. (a) Transverse section of kidney. Ventral is at bottom. Renal artery with ascending branch (ra1) which bends over into a horizontal course (ra2). Note the narrow spacing of afferent (glomerular) arterioles (arrows). (b) Afferent arteriole branching off a renal artery. Note the vascular sphincter at the origin of the afferent arteriole (arrows). Note also a ciliated peritoneal funnel aside the adventitia of a renal artery opening into a peritubular vessel (arrowhead). (c) Course and branching patterns of urogenital and renal arteries. Ventral view at an insufficiently cast right kidney. VCC. Scanning electron microscopy (SEM) micrograph. Rostral is at the left, lateral is at the bottom. Note the lack of any regular patterning of renal arteries. (d) Origin of urogenital arteries from the ventral aspect of the dorsal aorta. VCC. SEM micrograph. Arrow marks the origin of the celiac‐mesenteric artery. Inset 1: Closer view at the origin of three urogenital arteries from the dorsal aorta. Inset 2: Closer view at the origin of a genital artery from a urogenital artery. Note the triangular profile of the genital artery and the imprint of a flow divider on the vascular cast (arrow). Abbreviations: aa, afferent arteriole; Bc, Bowman's capsule; cma, celiac‐mesenteric artery; da, dorsal aorta; ga, genital artery; lda, left dorsal aorta; pcv, posterior caval vein; pf, peritoneal funnel; ra, renal artery; rda, right dorsal aorta; rpv, renal portal vein; uga, urogenital artery; VCC, vascular corrosion cast

In transverse sections of kidneys, the posterior caval vein, renal portal veins and their primary (first‐order branches) displayed the largest profiles followed by the ventrally located profiles of renal veins and arteries (Figure 2a). Afferent arterioles of glomeruli branched off from the renal arteries within short distances (Figure 3a). At the branching sites, a single layer of vascular smooth muscle cells composed muscular sphincters (arrows in Figure 3b). Generally, one afferent arteriole entered the vascular pole of a glomerulus and formed the interconnected wide glomerular capillaries that drained through one efferent arteriole into peritubular vessels.

### 3.1 Vascular anatomy

#### 3.1.1 Arterial supply

**FIGURE 4:**
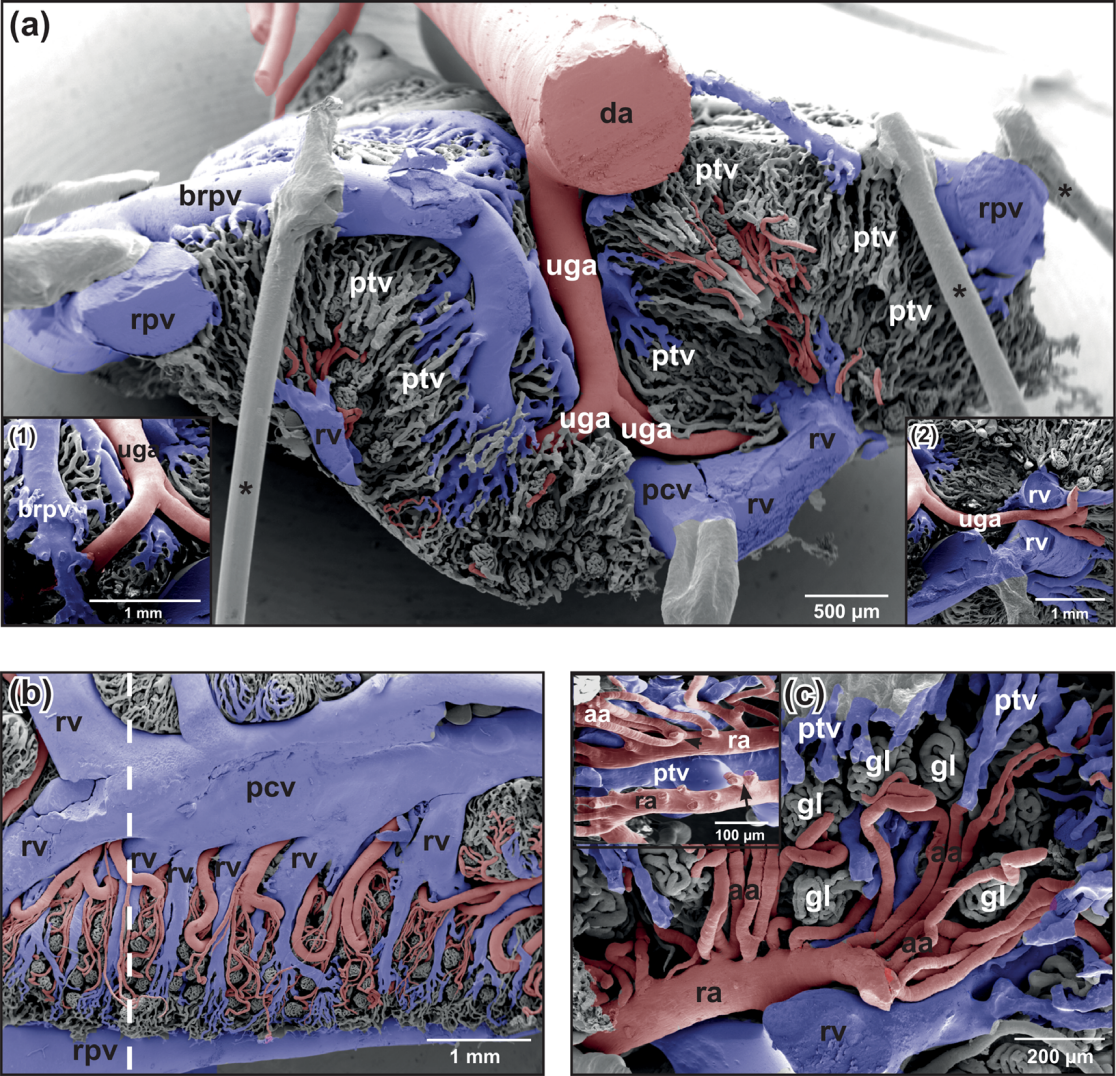
*Xenopus laevis*, microvascular anatomy of the kidneys of adult individual. VCCs. Scanning electron microscopy (SEM) micrographs. (a) Transverse section. Note a urogenital artery descending between kidneys and bifurcating toward right and left kidney. Inset 1: Spatial relationship between urogenital artery and a branch of the renal portal vein. Inset 2: Same, but between urogenital artery and renal veins. (b) Venous drainage of the kidneys. Ventral view. Note numerous renal veins draining into the posterior caval vein. Dashed line marks level of section displayed in Figure 5b. rpv, renal portal vein. (c) Renal artery with numerous afferent arterioles. Note that afferent arterioles primarily branch off as single individual vessels which feed nearby glomeruli or originate with a common stem to bifurcate after a short distance into single afferent arterioles. Inset: Intraparenchymal portion of renal arteries. Note that afferent arterioles arise as triplets (arrowhead) or even quadruplets (arrow) from the parent artery. Abbreviations: aa, afferent arteriole; brpv, branch of renal portal vein; da, dorsal aorta, gl, glomerulus; pcv, posterior caval vein, ptv, peritubular vessel, ra, renal artery; rpv, renal portal vein; rv, renal vein; VCC, vascular corrosion cast; uga; urogenital artery

**FIGURE 5:**
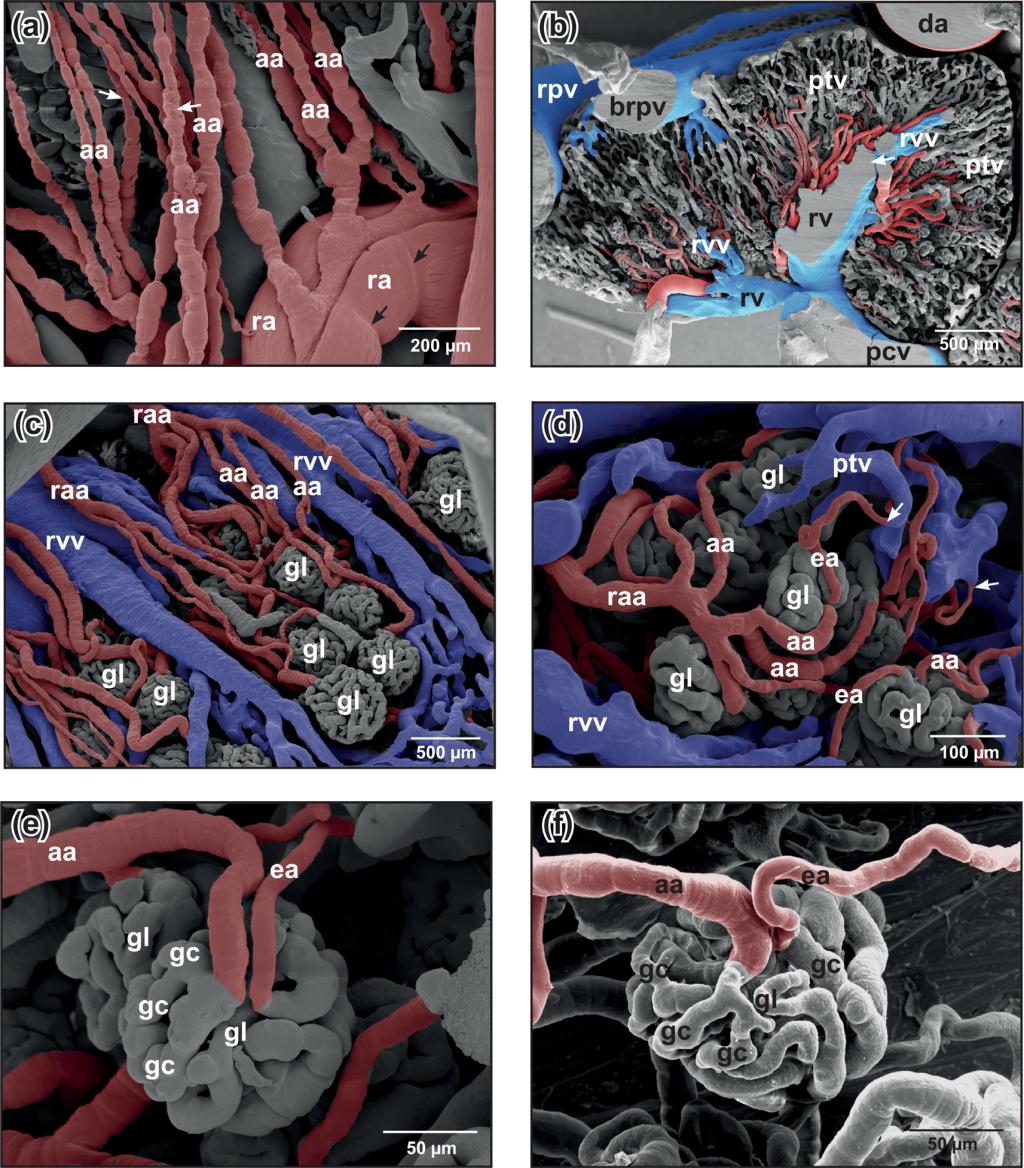
*Xenopus laevis*, microvascular anatomy of the kidneys of adult individual. VCCs. Scanning electron microscopy (SEM) micrographs. (a) Afferent arterioles with pearl‐string‐like appearance (small white arrows). Note circular imprints of vascular smooth muscle cells around renal arteries (black arrows). (b) Drainage of peritubular vessels into renal venules and renal veins. Transverse sectioned vascular corrosion cast. Note renal vein (arrow) which forms deep inside the kidney. (c) Afferent arterioles which continuously branch off a renal arteriole to supply more distal glomeruli. (d) Tree‐like branching pattern of a renal arteriole into many afferent arterioles. Note that efferent arterioles empty into peritubular vessels (arrows). (e) Glomerulus with afferent and efferent arteriole. Laterodorsal view at the vascular pole. The caliber of the afferent arteriole is larger than that of the efferent arteriole. Note that the efferent arteriole departs at about the same level as the afferent arteriole arrives. Glomerular capillaries anastomose frequently. (f) Glomerulus of an insufficiently cast kidney. Laterodorsal view at the vascular pole. Note the difference in the calibers of afferent arteriole and efferent arteriole. The efferent arteriole arises from deeper within the glomerulus. Abbreviations: aa, afferent arteriole; brpv, branches of the renal portal vein; da, ea, efferent arteriole; dorsal aorta; gc, glomerular capillary; gl, glomerulus; ptv, peritubular vessel; ra, renal artery; raa, renal arteriole; rpv, renal portal vein; rv, renal vein; rvv, renal venule; VCC, vascular corrosion cast

Six to eight urogenital arteries that branched in an obtuse angle from the ventral surface of the dorsal aorta descended along the medial margins of the kidneys (Figure 3c,d). They revealed prominent flow dividers at their origin (Figure 3d, insets 1,2). Renal arteries branched off already close to their origin (Figure 3d, inset 2) or at deeper levels (Figures 3c and 4a). Above the posterior caval vein, urogenital arteries occasionally bifurcated into a left and a right trunk, or coursed unbranched toward the ventral surface of the right or left kidney (Figure 4a). Most urogenital arteries bifurcated at the ventral surface of the kidneys into a smaller genital and a larger renal artery. Close to the ventral kidney regions, renal arteries gave off afferent (glomerular) arterioles (Figures 4b,c and 5a–c). Spacing, lengths, calibers, interbranching distances, and courses of afferent arterioles varied greatly (Figure 5a–d). In some renal casts, afferent arterioles had a prominent pearl‐string‐like appearance (Figure 5a). Bifurcations, but also a few trifurcations or even quadrifications (Figure 4c, inset) generally occurred after a short straight proximal portion, but sometimes also after a long unbranched portion (Figures 4c and 5a). Afferent arterioles splitted into glomerular capillaries that were slightly (Figure 5e) or conspicuously thinner (Figure 5f) than afferent arterioles. Glomerular capillaries were strongly interconnected, displayed outpouchings and narrowings, and were densely packed (Figure 5c–f) giving the glomeruli a spherical to slightly ovoid shape. Glomeruli drained via efferent arterioles into peritubular vessels (Figure 5d, arrows). In general, a single efferent arteriole left the glomerulus in close apposition to an incoming thicker afferent arteriole (Figure 5e,f). Efferent arterioles ascended toward dorsal and drained at varying levels into peritubular vessels (venules) (Figure 5d).

#### 3.1.2 Venous supply

**FIGURE 6:**
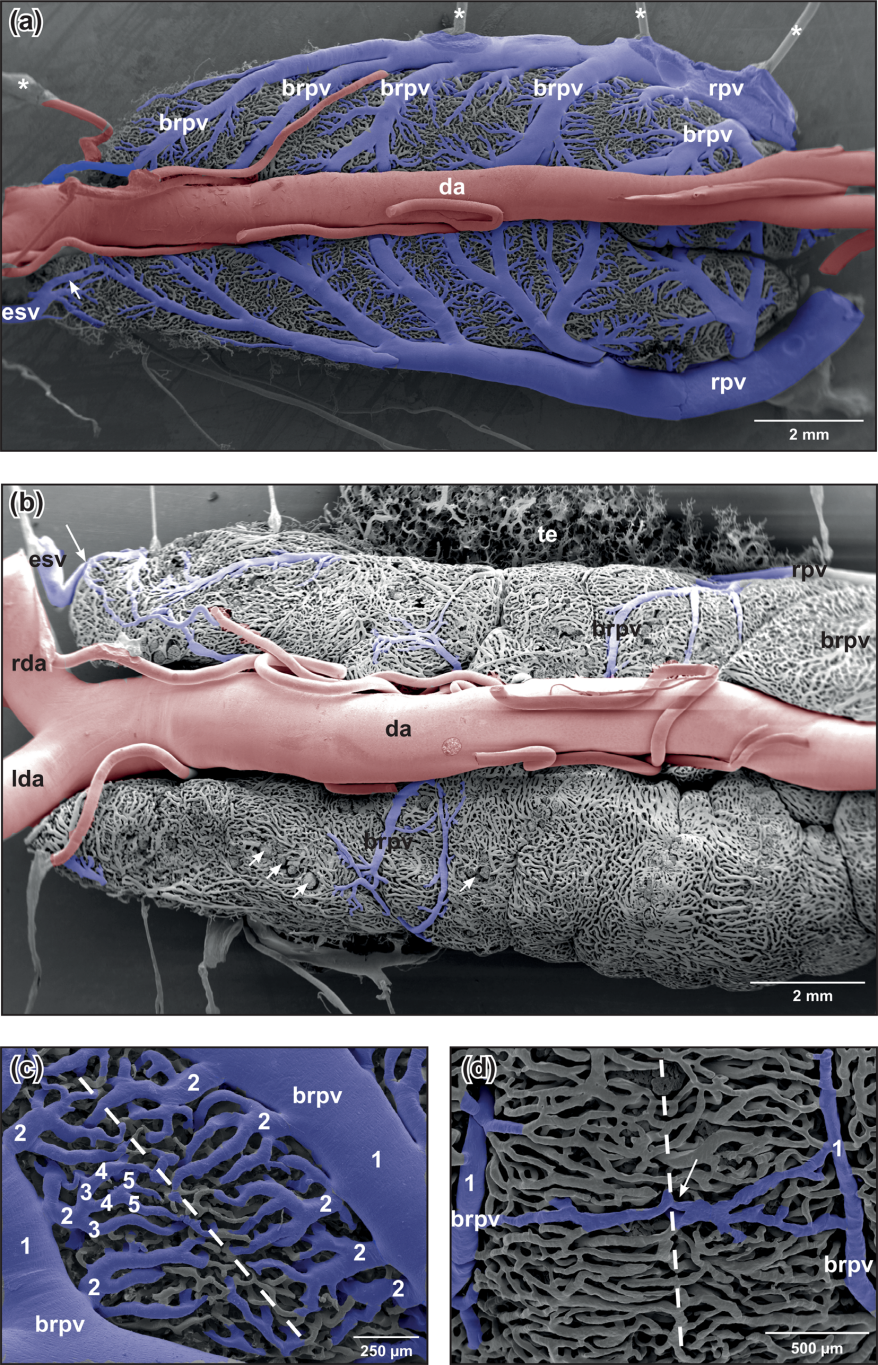
*Xenopus laevis*, microvascular anatomy of the kidneys of adult individual. Dorsal views. VCCs. Scanning electron microscopy (SEM) micrographs. Rostral is to the left. (a) The prominent renal portal veins course along the lateral margins of the kidneys toward rostral thereby giving off primarily rostromedially directed first‐order branches (brpv). Arrow points at an anastomosis between esophageal vein and renal portal vein. Asterisks mark conductive bridges. (b) Microvascular pattern of the dorsal surface of the kidneys. Note few and rather small first‐order branches (brpv) of the renal portal vein. Large arrow points at an anastomosis between esophageal vein and renal portal vein Small arrows indicate rather superficially located glomeruli. (c) Branching pattern of first‐order branches (brpv; 1) of the renal portal vein. Note up to four more branching orders (second–fifth) until capillary calibers are gained midway between consecutive first‐order branches (dashed line). (d) First‐order branches of the renal portal vein (brpv; 1) with rather uniform calibers. Note an anastomosis (arrow) which interconnects ipsilateral first‐order branches of the renal portal vein (brpv 1). Abbreviations: brpv, branch of renal portal vein; da, dorsal aorta; esv, esophageal vein; lda, left dorsal aorta; rda, right dorsal aorta; rpv, renal portal vein; te, testis; VCC, vascular corrosion cast

**FIGURE 7:**
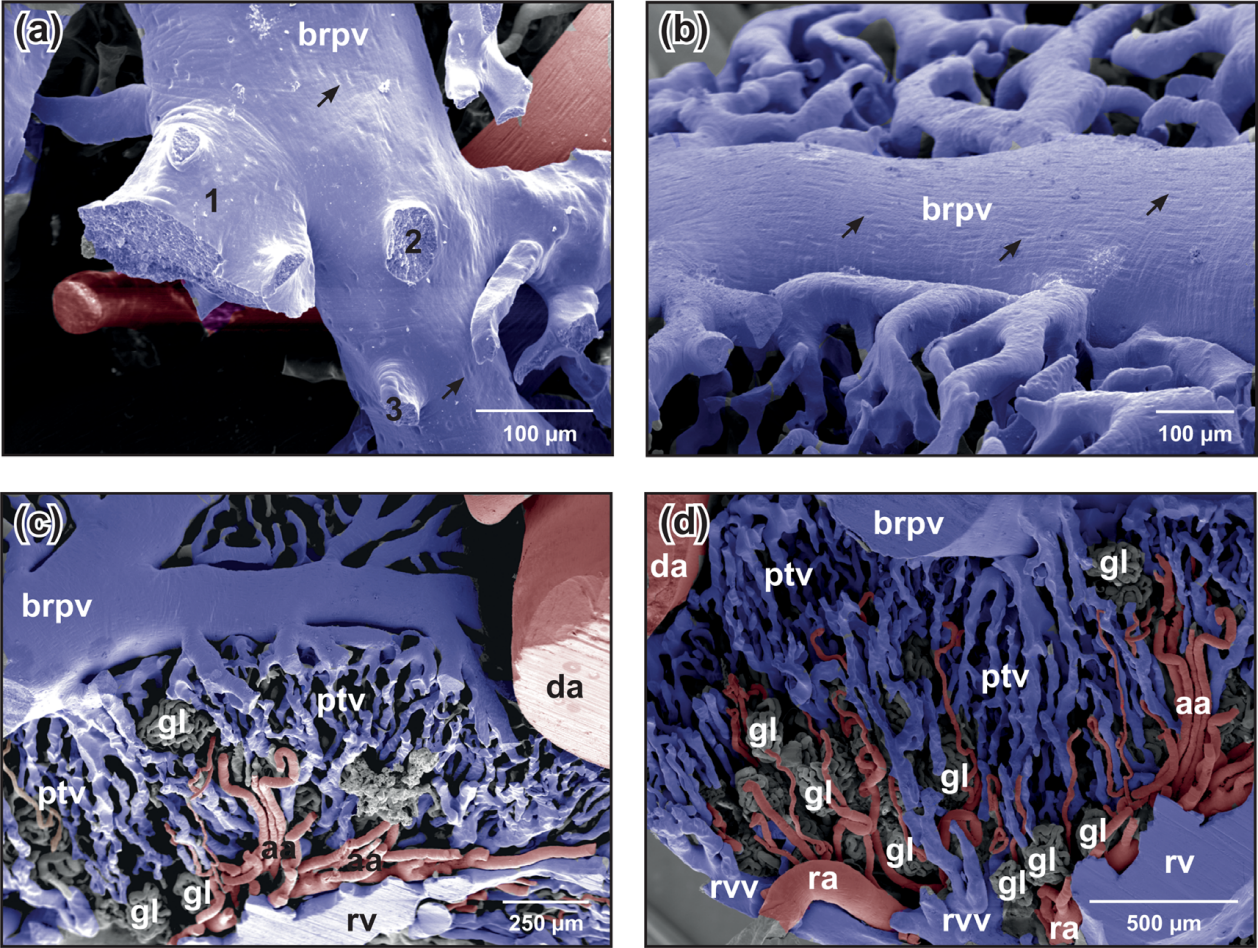
*Xenopus laevis*, microvascular anatomy of the kidneys of adult individual. VCCs. Scanning electron microscopy (SEM) micrographs. (a) First‐order branch of the renal portal vein displaying endothelial cell nuclei imprints characteristic of an artery (arrows). Note different calibers of side branches (1–3). (b) Endothelial cell nuclei imprints on a first‐order branch of the renal portal vein Note the longish imprints (arrows) orientated parallel to the long axis of the vessel characteristic of an artery. (c) Transition of fourth‐ or fifth‐order branches of the renal portal vein into peritubular vessels. (d) Transition of peritubular vessels into renal venules and renal veins. Abbreviations: aa, afferent arteriole; brpv,(first‐order) branch of renal portal vein; da, dorsal aorta; gl, glomerulus; ptv, peritubular vessel; ra, renal artery; rv, renal vein; rvv, renal venule; VCC, vascular corrosion cast

The mesonephric kidneys of *X. laevis* has a second source of blood supply via renal portal veins (Venae renales advehentes, Venae Jacobsonii) (Figure 6a). These veins formed from ischiatic, femoral, anterior and posterior dorsolumbar veins, and esophageal veins (Venae renales advehentes secundariae). Renal portal veins approached the kidneys at their latero‐posterior edges, coursed along their dorsolateral margins toward anterior, and gave off large side‐branches (Venae renales advehentes) that we termed first‐order branches. First‐order branches were primarily directed rostromedially (Figure 6a). Their calibers, numbers and courses differed greatly (Figure 6a,b). They gave off rostromedially and caudomedially directed branches of different calibers (=second‐order branches). These vessels lastly branched into third‐, fourth‐, and fifth‐order vessels (Figure 6c). Branchings into capillary sized vessels occurred within a short distance. Occasionally, branchings of first‐order vessels were inconspicuous (Figure 6b,d). In cases where first‐order branches were large, they extended over the laterodorsal surface and descended along the medial margins of the kidneys, where they displayed the same branching patterns as dorsal (Figure 4a). Second‐order branches had different calibers (Figure 7a) and displayed—like the renal portal veins—oval to longish endothelial cell nuclei imprints orientated parallel to the longitudinal axis of the vessel (Figure 7a,b).

First‐ to fifth‐order branches of the renal portal veins gave off branches in almost acute angles into the renal parenchyma (Figure 7c,d). These vessels further branched and formed the peritubular vessels (venules) which radiated rather straight toward ventrolateral or medioventral (Figures 4a and 7c,d). Peritubular vessels revealed irregular contours, ran mostly parallel, and anastomosed frequently. Locally they were flat, but often they abruptly changed into columnar with imprints of different sizes and shapes (Figure 7a,b). No peritubular vessels with clear cylindrical shapes were present. In ventral kidney areas, peritubular vessels converged and formed the initial portions of renal veins (venae renales revehentes) (Figures 4a and 7c,d).

#### 3.1.3 Venous drainage

**FIGURE 8:**
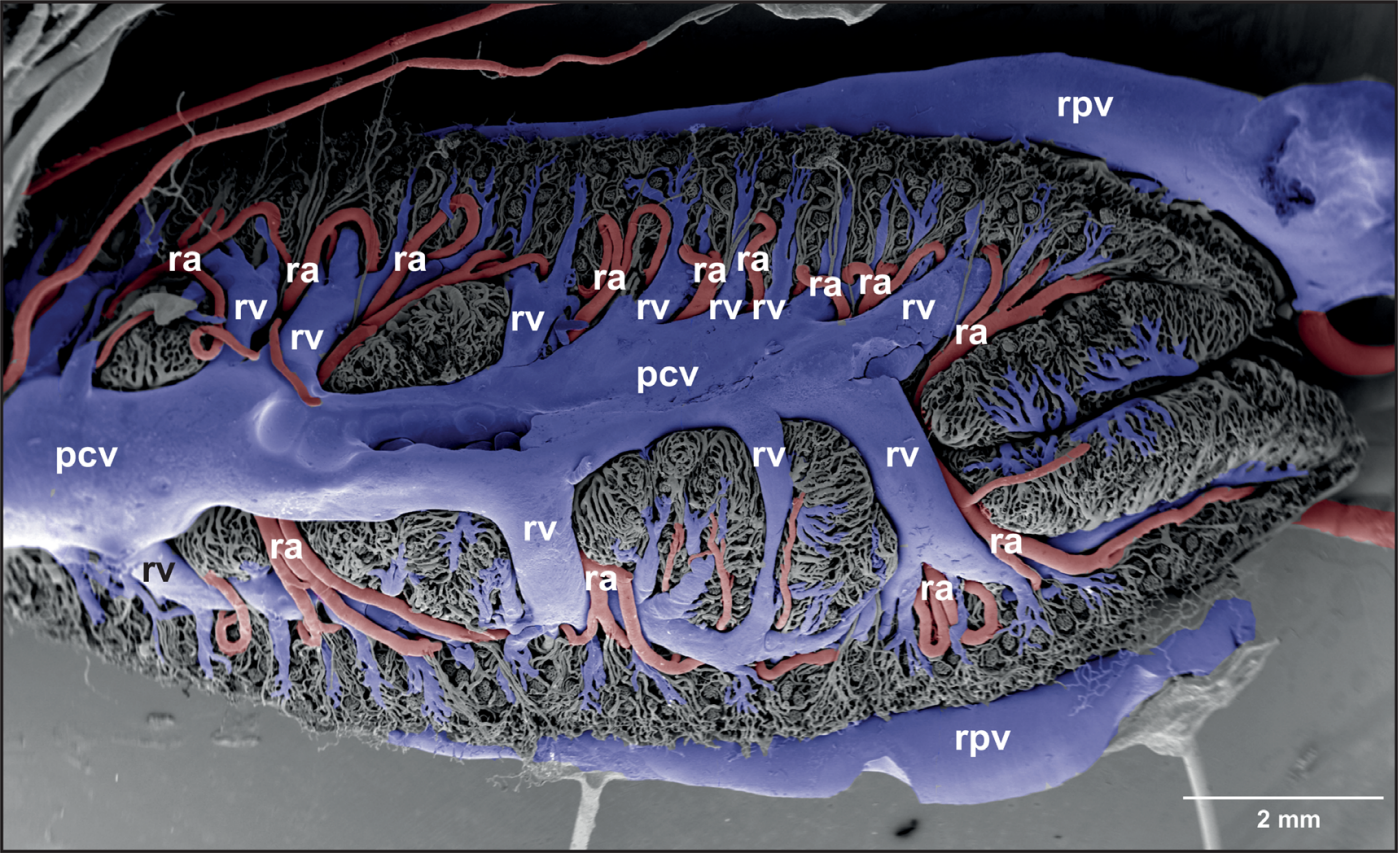
*Xenopus laevis*, microvascular anatomy of the kidneys of adult individual. VCC. Scanning electron microscopy (SEM) micrograph. Formation of the posterior caval vein by renal veins at the ventral surface of the kidneys. Abbreviations: pcv, posterior caval vein; ra, renal artery; rpv, renal portal vein; rv, renal vein; VCC, vascular corrosion cast

Kidneys drained via renal veins (venae renales revehentes) into the posterior caval vein that located ventrally between left and right kidneys (Figure 8). Numbers, calibers, courses and patterns of draining renal veins varied greatly between individuals and between right and left kidneys (Figures 4a,b, 7d, and 8). The shape of the posterior caval vein varied accordingly.

### 3.2 3D morphometry of vascular corrosion casts

Equatorial diameters of glomeruli ranged from 101 to 222 μm, distances from the vascular to the urinary pole were in the range from 54 to 173 μm. No significant differences were found between these diameters in glomeruli within kidney areas 1–8. Afferent glomerular arterioles showed diameters ranging from 15 to 36 μm whereas diameters of efferent glomerular arterioles ranged from 9 to 24 μm. For details, see Tables 1 and 2.

**TABLE 1:** Descriptive statistics of glomerular dimensions (in μm; ed = equatorial diameters; vud = vascular–urinary pole distance) within mediodorsal areas (1), mediomedial areas (2), medioventral areas (3), dorsomedial areas (4), dorsolateral areas (5), ventrolateral areas (6), ventromedial areas (7), and central areas (8) of kidneys in adult *X. laevis*. *N* = 103. For areas, see Figure 1

Parameter	*n*	Mean	*SD*	CI of mean	Median	Range	*Q*_25%_	*Q*_75%_
ed 1	5	153.5	19.2	23.9	148.5	46.9	137.0	172.5
ed 2	1	150.1	—	—	150.1	—	—	—
ed 3	7	131.6	24.7	22.9	135.0	74.5	112.7	143.3
ed 4	8	176.3	34.0	28.4	188.7	79.1	137.1	208.1
ed 5	2	167.0	40.9	367.5	167.0	57.8	138.1	195.9
ed 6	13	175.2	26.0	15.7	171.7	100.0	158.0	196.4
ed 7	8	161.3	30.8	25.7	159.4	90.8	140.3	193.2
ed 8	17	175.8	26.5	13.6	180.7	92.5	148.3	198.3
vud 1	5	145.0	18.2	22.6	149.8	37.0	125.8	161.7
vud 2	1	96.3	—	—	96.3	—	—	—
vud 3	7	106.9	13.4	12.4	106.7	36.0	91.4	116.7
vud 4	8	131.0	27.5	23.0	135.7	96.5	118.0	142.5
vud 5	2	112.8	3.9	35.3	112.8	5.5	110.0	115.5
vud 6	11	121.7	14.6	9.8	120.3	47.9	111.6	134.4
vud 7	8	97.5	21.9	18.3	97.5	72.5	88.2	113.5
vud 8	17	121.8	19.5	10.0	125.9	61.7	103.2	137.7

**TABLE 2:** Descriptive statistics of diameters (in μm) of afferent (aa) and efferent (glomerular) arterioles (ea) within mediodorsal areas (1), mediomedial areas (2), medioventral areas (3), dorsomedial areas (4), dorsolateral areas (5), ventrolateral areas (6), ventromedial areas (7), and central areas (8) of kidneys in adult *X. laevis*. *N* = 64. For areas, see Figure 1

Parameter	*n*	Mean	*SD*	CI of mean	Median	Range	*Q*_25%_	*Q*_75%_
aa 1	4	26.5	3.3	5.2	26.1	7.9	23.6	29.8
aa 2	1	21.8	—	—	21.8	—	—	—
aa 3	3	31.7	4.0	9.9	32.6	7.7	27.3	35.0
aa 4	7	24.5	3.8	3.5	23.5	10.7	20.7	27.3
aa 5	—	—	—	—	—	—	—	—
aa 6	7	30.4	4.0	3.7	28.9	10.3	27.0	35.4
aa 7	3	24.2	7.5	18.6	27.4	14.1	15.7	29.9
aa 8	9	23.8	7.2	5.5	21.9	20.9	17.4	30.5
ea 1	1	16.6	—	—	16.6	—	—	—
ea 2	1	15.1	—	—	15.1	—	—	—
ea 3	4	18.4	3.0	4.8	17.7	7.1	16.0	21.5
ea 4	5	17.6	3.4	4.2	18.3	9.3	14.6	20.3
ea 5	1	16.5	—	—	16.5	—	—	—
ea 6	6	16.5	5.5	5.8	16.3	15.6	11.8	21.5
ea 7	3	16.0	6.5	16.0	17.4	12.7	8.9	21.6
ea 8	9	13.6	2.6	2.0	13.7	8.3	11.9	15.6

## 4 DISCUSSION

Numerous studies describe the renal vascular anatomy in vertebrate taxa including men. The most detailed insights into the microangioarchitecture were gained when using SEM and nano‐computer tomography of vascular corrosion casts (Murakami, 1971; Wagner et al., 2011). Studies primarily focused upon mammalian (metanephric) kidneys, particularly rodent kidneys in healthy (e.g., Casellas, Mimran, Dupont, & Jover, 1981; Murakami, 1972, 1976; Wagner et al., 2011) and under experimental conditions (e.g., Nelson, Shah‐Yukich, & Barbaryan, 1984). In our casts, renal glomeruli were found throughout the entire kidney with most glomeruli located at the ventral half. This contrasts with previous findings in *R. catesbeiana* (Ohtani & Naito, 1980) and *B. marinus* (Morris & Campbell, 1978) where glomeruli were reported to locate at the ventral third of the organ.

In some of our renal casts, afferent (glomerular) arterioles revealed abundant circular imprints giving the arterioles a pearl‐string‐like appearance. Imprints most likely resulted from circularly arranged vascular smooth muscle cells (Figure 5a) which accidently contracted during the casting procedure. If the efferent arterioles, which lacked these imprints, had less vascular smooth muscle cells, or if vascular smooth muscle cells did not contract and cast efferent arterioles therefore lacked circular imprints could not be decided by SEM of vascular corrosion casts and remains open for further fine structural studies. The finding by Morris & Gibbins (1983) that in adult *B. marinus* “… every smooth muscle cell in the afferent arterioles is multiply innervated” and “…only 3‐5% of efferent arterioles are accompanied by single adrenergic nerve fibers”, might explain our observation providing the same patterns of innervation of renal vessels in *Xenopus*.

The abundant circular arrangement of vascular smooth muscle cells in afferent arterioles is of interest with respect to the tubuloglomerular feedback and the myogenic mechanism, which are reported to jointly act to autoregulate single‐nephron blood flow in mammals (Marsh et al., 2017). With the circularly arranged vascular smooth muscle cells in afferent glomerular arterioles in *Xenopus*, the structural basis for such a myogenic mechanism is given. The muscular sphincters found at the origin of afferent arterioles could also play an import role in the regulation of blood flow to individual renal glomeruli.

Marsh et al. (2017) analyzed the architecture of the nephron‐arterial network in resin casts of rat kidneys using microcomputed tomography. They described three pattern of origin of the afferent glomerular arterioles: (a) nonterminal (renal) arteries (Motif 1), (b) pairs or triplets of afferent arterioles arising from terminal (renal) arteries (Motif 2), and (c) unpaired branches of nonterminal (renal) arteries which end with a pair of afferent arterioles (Motif 3). Though gross renal blood supply in anurans and mammals differs substantially, there are some similarities with respect to the origin of afferent glomerular arteries, as we found in our specimens (a) afferent glomerular arterioles which arose from nonterminal renal arteries (Motif 1) (see Figure 4c), (b) afferent glomerular arterioles which arose pair‐wise from a terminal artery (Motif 2) (see Figure 5d), and (c) afferent glomerular arterioles which arose as pairs from the end of terminal arteries (Motif 3) (see Figure 5c).

In our earlier work on the vascularization of the kidneys in *B. bufo*, *B. variegata*, *R. ridibunda*, and *X. laevis* (Lametschwandtner et al., 1978), we neither focused specifically on the origins of afferent (glomerular) arterioles (i.e., Motifs 1–3), nor on the cast's surface morphology. We identified the resin‐filled (cast) dorsal aorta and the renal portal veins by comparing their origins and courses with those reported by Krause (1923). In the present study, we additionally identified arteries and veins by their characteristic endothelial cell patterns (shape, alignment) imprinted (replicated) on the surfaces of cast arteries and veins. The observation that casts of renal portal veins and their first‐order branches revealed endothelial surface imprint patterns described as characteristic for arteries by Miodonski et al. (1976), that is, endothelial cells and endothelial cell nuclei align parallel to the longitudinal axis of the artery, is not surprising. Such an alignment, which is considered to be induced by flow or shear stress and which protects the vascular wall from inflammation and permeability (Kroon et al., 2017), has been described in the rat hepatic portal vein (Dong, Ichimura, & Sakai, 2010). These authors also showed that in regions where the intimal folds of the hepatic portal vein disappeared, polygonal‐shaped endothelial cells, which were described by Miodonski et al. (1976) as characteristic for veins, were present. In case of portal veins, the identification of the nature of cast blood vessels solely by means of endothelial cell and endothelial cell nuclei imprint patterns on the cast's surfaces is misleading. The alignment of endothelial cells found in the renal portal veins and their first‐order branches in *Xenopus* most likely reflects high flow and shear stress in these vessels, a suggestion to be tested in future physiological experiments.

In the present study, we neither paid attention to the question if numbers and sizes of renal glomeruli differed between younger and older individuals, nor did we attempt to correlate specific vascular features, like the number of branchings of the renal arteries or of the renal portal veins with size or weight of individuals. To clarify these interesting questions in respect to postmetamorphic remodeling of the renal vasculature further work is needed.

## CONFLICT OF INTEREST

Both the authors declare no conflicts of interest.

## AUTHOR CONTRIBUTIONS

Alois Lametschwandtner performed resin injections, part of processing, and SEM analyses of vascular corrosion casts, analyses of tissues sections, and most figure colorations. Bernd Minnich performed the statistical analysis of 3D data. Alois Lametschwandtner and Bernd Minnich equally participated in drafting, critical revision, and final approval of the manuscript and the figure plates.

## Data Availability

The data that support the quantitative findings of this study are available from the corresponding author (Alois.Lametschwandtner@sbg.ac.at) upon request.
